# Congenital Caudal Vertebral Anomalies in a Pedigree of Beagles

**DOI:** 10.3390/ani16213373

**Published:** 2026-10-26

**Authors:** Sydney J. Fish, Jane M. Manfredi, Char L. Panek, Amanda L. Jacobson, Christine D. Harman, Paige A. Winkler, Keiko Miyadera, András M. Komáromy

**Affiliations:** 1 Department of Small Animal Clinical Sciences, College of Veterinary Medicine, Michigan State University, East Lansing, MI 48824, USA; fishsydn@msu.edu (S.J.F.); ande2133@msu.edu (A.L.J.); harmanc1@msu.edu (C.D.H.); winkle38@msu.edu (P.A.W.); miyadera@msu.edu (K.M.); 2 Department of Large Animal Clinical Sciences, College of Veterinary Medicine, Michigan State University, East Lansing, MI 48824, USA; panekcha@msu.edu

**Keywords:** radiographic examination, malformation, block vertebra, hemivertebra

## Abstract

Despite increased recognition of caudal vertebral anomalies in the domestic dog (*Canis lupus familiaris*), studies describing tail deformities remain limited. To address this gap in the literature, this study aimed to identify and classify the caudal vertebral anomaly phenotypes in a pedigree of Beagles. Subjects included 67 dogs (36 males and 31 females), aged eight months to eight years old (M= 38.8 ± 25.6 months). All 67 dogs were visually examined, and tails were palpated to determine if they had any kinks, bends, misalignments, or deformities in their tail prior to radiographic assessment. Radiographs of the caudal vertebrae were obtained from 53 dogs and evaluated for congenital anomalies, including block vertebrae, hemivertebrae, brachyury (very short tail), and axial deviations. Of the 53 radiographed dogs, 25 (47.2%) exhibited at least one congenital caudal vertebral anomaly, with block vertebrae being the most common finding (43.4%). Four dogs (7.6%) had mild congenital abnormalities detected radiographically that were not identified during physical examination. Based on pedigree structure, an autosomal recessive trait is suspected. These findings demonstrate a high prevalence of caudal vertebral anomalies within this pedigree of Beagles and emphasize the importance of radiographic evaluation for accurate identification and phenotypic classification.

## Simple Summary

Since restrictions on cosmetic tail docking have become more widespread, congenital caudal vertebral abnormalities and naturally occurring tail deformities have received increased attention. However, few studies characterize congenital tail deformities in dogs. The present study aimed to identify and classify the tail deformity phenotypes through physical and radiographic examination in a pedigree of Beagles. A total of 67 dogs (36 males and 31 females), aged 8 months to 8 years old, were visually examined, and tails were palpated to determine if they had any kinks, bends, or misalignments in their tails prior to radiographic assessment. Radiographs of the tail (caudal vertebral column) were obtained from 53 dogs and evaluated for congenital deformities. Of the 53 radiographed dogs, 25 (47.2%) exhibited at least one deformity of the caudal vertebrae, with the most common finding being the fusion of vertebral bodies (block vertebrae). Four dogs (7.6%) had mild congenital abnormalities detected radiographically that were not identified during physical examination. These findings demonstrate a high prevalence of caudal vertebral anomalies within this family of Beagles and emphasize the importance of radiographic evaluation for accurate identification and phenotypic classification.

## 1. Introduction

The canine (*Canis lupus familiaris*) tail contains a variable number of vertebrae that assist in balance and communication. Dogs typically have approximately 20 caudal vertebrae, although the past literature has reported a range of six to 23 [1]. Kink tail is a trait that the breeders’ community discusses as a reason to exclude a dog from use for breeding purposes [2]. Morphological differences have been a major driver of selective dog breeding since domestication and the formation of breeds [3]. Morphological traits may also be genetically linked to disease-predisposition traits [4,5]. Therefore, it is important to determine specific phenotypic variations, as it can provide insight into disease origins.

Congenital anomalies of the caudal vertebrae are relatively common incidental findings in dogs and include hemivertebrae and block vertebrae [1,2]. Block vertebrae are defined as the fusion of two or more adjacent vertebral bodies, and hemivertebrae occur when only one side of the vertebral body forms properly, leaving a wedge-shaped vertebra and often causing an axial deviation in the tail [6]. Hemivertebrae are defined as a failure of the formation of a vertebral body, usually resulting in a wedge-shaped vertebra with the base oriented dorsally, ventrally, or medially [6]. Block vertebrae are a congenital anomaly that develops during the fetal period, which is distinct from transitional and butterfly vertebrae, which develop during the embryonic period [7]. Butterfly vertebrae result from a lack of fusion of the two lateral ossification centers, leaving two adjacent hemivertebrae, or the appearance of a single vertebra divided vertically through the center [6,7]. In dogs, hemivertebrae are seen most frequently in the screw-tailed breeds (Bulldog, French Bulldog, Pug, Boston Terrier) because the kinked tail is due to caudal hemivertebrae [8]. Block vertebrae have been documented in human studies as early as 1894 and are most commonly reported in the cervical and lumbar regions [6]. In dogs, these anomalies may be detected through visual or physical examination depending on their severity and may also affect the caudal vertebrae. Some affected dogs do not exhibit an obvious kink or axial deviation in the tail, and anomalies may only be detected through radiographic examination. In most cases, caudal vertebral malformations are not clinically significant and do not cause any pain or other health-related issues [6].

Radiographic assessment remains the primary method for classifying caudal vertebral anomalies in dogs. In a radiographic study of 595 dogs, congenital caudal vertebral anomalies (including block vertebrae) were identified in 11.09% of the population examined, with the highest prevalences being in the Belgian Shepherd Dog, Bernese Mountain Dog and Hovawart [1]. The prevalence of congenital vertebral malformations varies considerably among breeds, with screw-tailed brachycephalic breeds being particularly predisposed. For example, in a previous study on 171 neurologically normal and 10 neurologically abnormal screw-tailed brachycephalic dogs, four of 62 French Bulldogs (6.5%) and four of 41 English Bulldogs (9.8%) had caudal thoracic block vertebrae [9]. In that study, block vertebrae occurred in the neurologically normal group, whereas the thoracic vertebral malformations that caused neurological dysfunction in the abnormal group were vertebral body formation defects [9]. Another study consisting of 149 screw-tailed dogs identified lumbosacral hemivertebrae in 32.0% of French Bulldogs, 24.3% of English Bulldogs, and 1.7% of Pugs [10].

Previous investigations have revealed differences in the type, location, and severity of vertebral malformations among individuals and breeds, as well as a classification of anomalies based on radiographs [1,2]. Beyond phenotypic characterization, the past literature has also identified genetic factors that may contribute to the development of caudal vertebral anomalies. The C189G mutation in exon 1 of the T-box transcription factor *T* gene (*TBXT)* affects the DNA-binding ability of the T protein due to the substitution of Met for Ile at residue 63, resulting in naturally occurring short tail phenotypes in multiple breeds, including the Pembroke Welsh Corgi, Jack Russel Terrier, and White Swiss Shepherd [10,11,12]. The *TBXT* gene is a member of the T-box family of transcription factors consisting of more than 20 genes that are involved in embryogenesis and the development of axial structures, including the tail [13]. In mice, mutations in the T-gene are associated with skeletal defects in heterozygotes, whereas homozygous mutants have severe developmental disorders and do not survive [10,14].

Vertebral development is regulated by several molecular pathways that coordinate somitogenesis, segmentation, and subsequent skeletal formation. A frameshift deletion variant in the dishevelled segment polarity protein 2 (*DVL2* gene) has been associated with caudal vertebral malformations, including block vertebrae and hemivertebrae, in breeds with a screw-tail trait (English Bulldogs, French Bulldogs, and Boston Terriers) [15]. These breeds also exhibit brachycephalic skull morphology, widely set eyes, and short stature, which resemble traits of Robinow syndrome in humans, a congenital disorder associated with variants in genes involving the Wnt signaling pathways, including *DVL1* and *DVL3* [16]. Wnt signaling occurs through the canonical pathway, which regulates cell fate determination, and the noncanonical pathway, which regulates cell movement and tissue polarity [17]. In mouse models, the *Pax1* and *Wnt-3a* genes have been found to influence vertebral development, leading to shortened or kinked tails in some cases [18,19]. Genes involved in the Notch signaling pathway have also been associated with vertebral malformations in dogs. A mutation in *HES7* has been identified in Miniature Schnauzers with spondylocostal dysostosis and hemivertebrae [20]. *HES7* is involved in the segmentation clock that regulates somitogenesis, and disruption of its function can interfere with normal somite formation and subsequent vertebral segmentation [20]. Although a specific gene associated with block vertebrae has not been identified, these findings suggest that disruptions in pathways involved in somitogenesis and vertebral segmentation may contribute to abnormal vertebral development and fusion.

Despite increased recognition of caudal vertebral anomalies in dogs, detailed phenotypic descriptions remain limited. Characterization of these traits within specific pedigrees provides an overview of the spectrum of phenotypes present and identifies patterns that may warrant further research in related genetic anomalies. The objective of this study was to identify and classify the caudal vertebrae phenotypes in a pedigree of purpose-bred Beagles. This work aimed to describe the caudal vertebral phenotypes within this family of dogs and to lay the foundation for future investigations into their genetic basis. By providing a characterization of these anomalies within a defined pedigree, this study expands the current understanding of the prevalence, type, and severity of caudal vertebral abnormalities in this family of Beagle dogs.

## 2. Materials and Methods

### 2.1. Animals

Subjects included 67 dogs (36 males and 31 females), aged 8 months to 8 years old (M = 38.8 ± 25.6 months), that are part of a group of Beagles that contains animals with naturally occurring open-angle glaucoma caused by a G661R missense mutation in *ADAMTS10* developed in 1972 and now maintained at Michigan State University [21,22]. The *ADAMTS10* genotype was included to investigate a potential association between the G661R missense mutation and the occurrence of caudal vertebral abnormalities within this pedigree. *ADAMTS10* genotypes for each dog were confirmed by gene sequencing using methods described by [22]. All procedures were approved by the Institutional Animal Care and Use Committee; see Institutional Review Board Statement for details.

### 2.2. Pedigree Construction

A pedigree was constructed from breeding records, and affected dogs were designated on the basis of their clinical findings. Phenotypic classification, based on physical examination (*n* = 67) and/or radiographic examination (*n* = 53), was indicated as categorized below from mild, moderate, to severe in the affected animals. The likely mode of inheritance was evaluated under the assumption of a simple Mendelian trait, provided that segregation of the phenotype within the pedigree was consistent with this model.

### 2.3. Physical Examination

All 67 dogs were visually examined at birth and at the time of the study for dogs who were still present (*n* = 53), and tails were palpated to determine if they had any kinks, bends, misalignments, or deformities in their tail prior to radiographic assessment. The entire tail was palpated from the base to the tip to identify any palpable abnormalities of the caudal vertebral column.

### 2.4. Radiographic Evaluation

Following physical examination, radiographs of the caudal vertebrae were obtained for 53 dogs. The remaining 14 dogs were unavailable for radiographs (either deceased or adopted) and were therefore phenotyped based solely on physical examination. Radiographs were taken using a Sprint DR III Elite Equine machine (Antech Diagnostics, Carlsbad, CA, USA) at a setting of 60 kV and 0.50 mAs. Dogs were sedated with dexmedetomidine (2–3 mcg/kg, intravenous; Dechra Ltd., Overland Park, KS, USA) and butorphanol (0.2 mg/kg, intravenous; Dechra Ltd., Overland Park, KS, USA) to obtain a clear image. Laterolateral and ventrodorsal views of the entire caudal spine were obtained. Laterolateral views were used for statistics due to being able to visualize all vertebrae. Dogs were then given atipamezole (0.02–0.03 mg/kg, intramuscular; Antisedan; Zoetis Inc., Parsippany, NJ, USA) to reverse the sedation and were allowed to recover from sedation under close supervision.

Images of all the caudal vertebrae were reviewed using an online browser-based DICOM interface to identify any abnormalities that were either clinically apparent or not detectable during physical examination [23]. Abnormalities were differentiated from acquired changes based on the radiographic classification described by Paninárová et al. [1], in which hemivertebrae and block vertebrae are classified as congenital abnormalities, while fractures, callus formation, and luxation are classified as post-traumatic changes. Spondylosis, osteophyte formation, and intervertebral mineralization were classified as degenerative changes [1]. No radiographic evidence of previous fracture or other post-traumatic changes was identified in dogs classified as having congenital abnormalities. A single observer (SJF) conducted all radiographic reviews to minimize interobserver variability.

### 2.5. Phenotypic Classification

**Figure 1:**
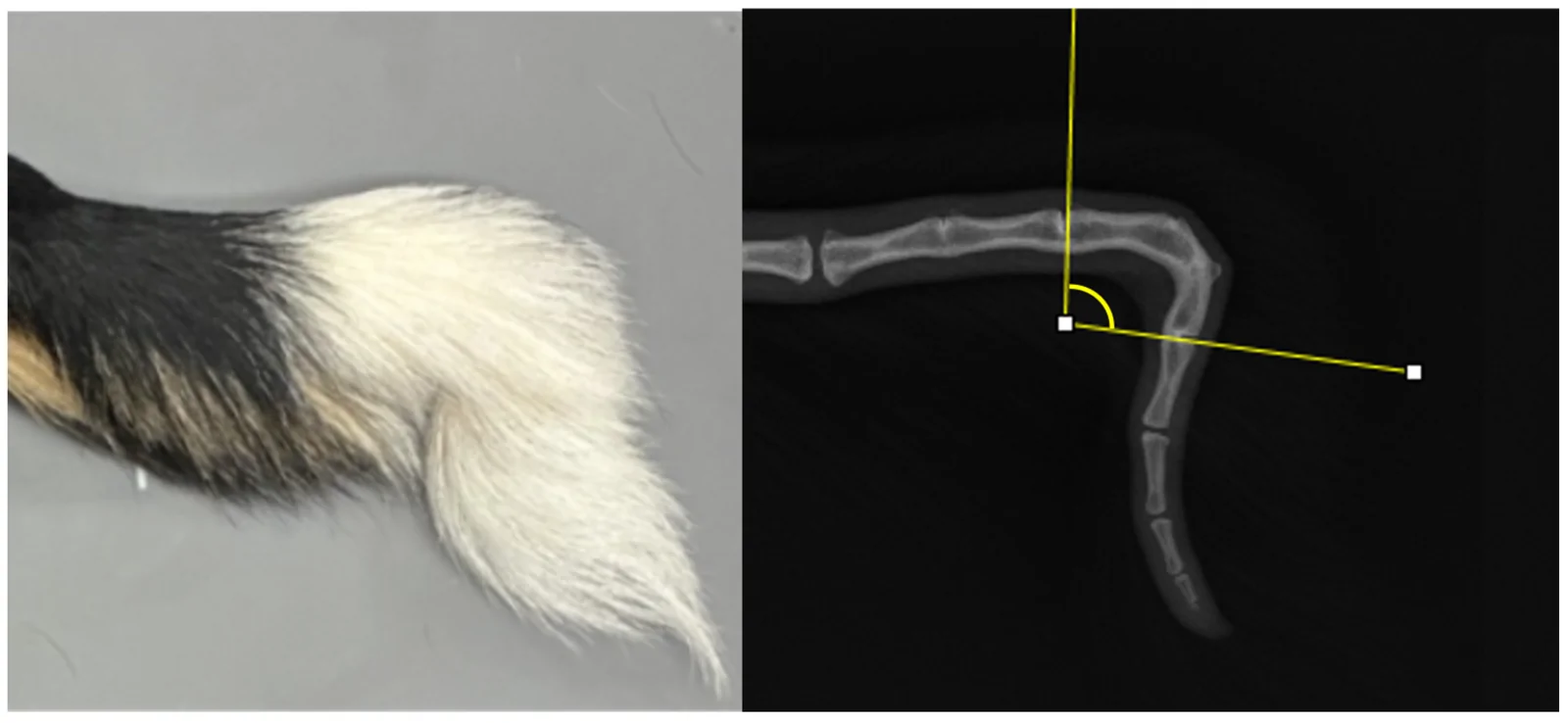
Example of Cobb angle measurement in a dog. A gross external image (**left**) and a corresponding radiograph used to calculate the Cobb angle of the axial deviation (yellow lines on the **right**) are shown.

Tail phenotypes were assessed and scored as mild, moderate, or severe using a novel scoring system (described below). Vertebral abnormalities were classified based on previously described criteria [1,24]. The factors taken into consideration when classifying each tail included the total number of caudal vertebrae, the location, number and type of affected vertebrae, and the angle of the bend(s) in the tail (Figure 1). Angles of the bends in each tail were measured manually using FIJI Software (Windows version x86-64, Java). The angles were measured using the Cobb angle, which has been used in previous studies to quantify the degree of spinal curvature in dogs and humans [25,26]. The Cobb angle is the angle formed between a line parallel to the superior endplate of the uppermost vertebra and the inferior endplate of the lowermost vertebra involved in the deviation [26]. Axial deviation was defined as an immovable deviation of the tail from the medians. Block vertebrae were defined as fusion between two or more adjacent vertebrae, with loss of clearly visible boundaries between the affected vertebrae [27,28,29]. Brachyury was defined as a naturally very short tail, having fewer caudal vertebrae than normal. Lastly, hemivertebrae were defined as a wedge-shaped bony structure between two adjacent vertebrae.

### 2.6. Determining Locations of Deformities

The location of each deformity was identified by counting the individual vertebrae in the tail and determining where the fusion or deviation begins. The caudal vertebral column was divided into cranial, middle, and caudal thirds based on the total number of caudal vertebrae present in each dog. For example, in a dog with 21 caudal vertebrae, Cd1–Cd7 were classified as the cranial third, Cd8–Cd14 as the middle third, and Cd15–Cd21 as the caudal third.

### 2.7. Novel Scoring System

**Figure 2:**
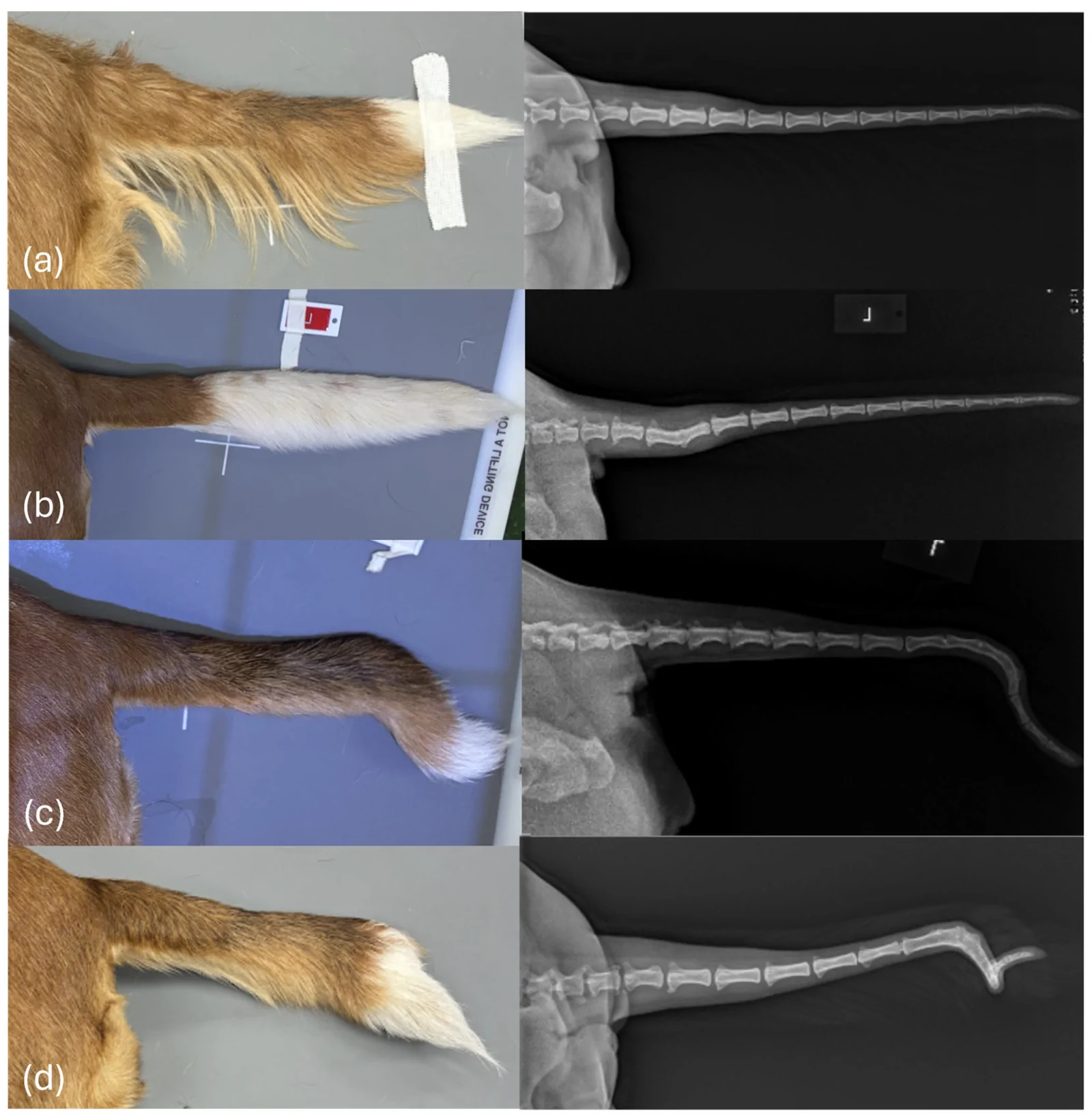
Examples of varying severity in caudal vertebral anomalies: the external view (**left**) and corresponding radiographs (**right**) are shown. (**a**) Normal dog (#43 Uni1); (**b**) mildly affected (#24 Yak); (**c**) moderately affected (#10 Tac); (**d**) severely affected (#1 Udo).

Tail phenotypes were scored based on a novel scoring system (Table 1) that was developed to categorize caudal vertebral anomalies as mild, moderate, or severe (Figure 2). No literature known to the authors has described a method to categorize variability in caudal vertebrae. The previous literature has described caudal vertebral changes, and elements of their characteristic descriptions, including block vertebrae and hemivertebra, are included in this scheme [1,26]. In addition, we have included angulation measurements of the tail to help quantify the severity of the deviation. Describing the severity of affectedness of caudal vertebral abnormalities could be important for correlating phenotype to genotype. To that end, we looked at the reported number of block vertebrae in dogs in previous and this current study to determine a range for mild, moderate, and severe anomalies. Cobb angle was performed manually as described in Chen et al. [26]. Brachyury and hemivertebrae were each assigned one point to account for their presence as distinct structural abnormalities. Finally, the total score ranges were divided into mild (1–2), moderate (3–4), and severe (5–7) categories to provide a standardized method for describing the spectrum of phenotypic severity observed within this population. These thresholds are defined for study purposes and are intended for phenotypic classification rather than validated clinical severity cutoffs.

The scoring system incorporated three characteristics of the tail phenotype: number of fused caudal vertebrae, degree of tail angulation, and presence of brachyury and/or hemivertebrae. Each characteristic was assigned a numerical score according to its severity, and individual scores were summed for a final deformity score for each dog. The number of fused vertebrae was scored as zero points for zero to one fused vertebra, one point for two to three fused vertebrae, two points for four to eight fused vertebrae, and three points for nine or more fused vertebrae (Table 1). Axial deviation was evaluated using the Cobb angle at up to two distinct deviations from the medians along the tail [26]. Each bend was measured and scored independently according to its degree of angulation. A straight tail (0°) received zero points, an angle of >0° to 60° received one point, and an angle of >60° received two points (Table 1). When two bends were present, the score for each bend was included separately in the final score. The presence of brachyury or hemivertebrae was also assigned a single point (Table 1). Dogs with a final score of one to two were classified as having a mild deformity; three to four were classified as moderate, and five to seven were classified as severe.

### 2.8. Data and Analysis

Descriptive statistics were performed to evaluate the total number of caudal vertebrae, the angle(s) of the bend in the tail, and the number of block vertebrae. Frequencies of congenital anomalies within the population were also calculated.

## 3. Results

### 3.1. Radiographic Examination Findings

**Figure 3:**
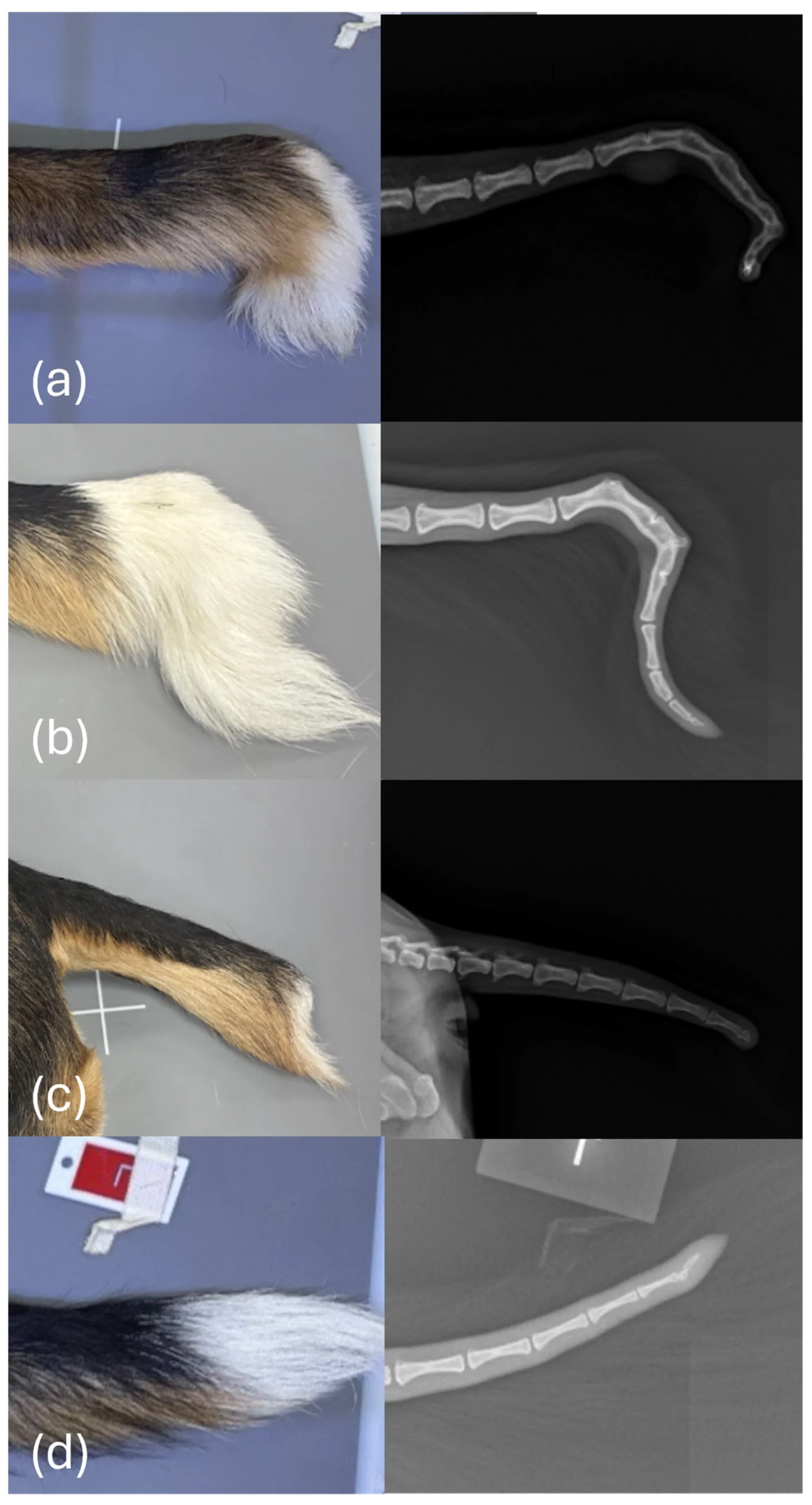
Examples of congenital anomalies of the caudal vertebrae: the external view (**left**) and corresponding radiographs (**right**) are shown. (**a**) block vertebrae and axial deviation (#4 Zuc); (**b**) block vertebrae and axial deviation (#16 Zuk); (**c**) brachyury (#21 UFO); (**d**) hemivertebrae (#18 Cor2).

**Figure 4:**
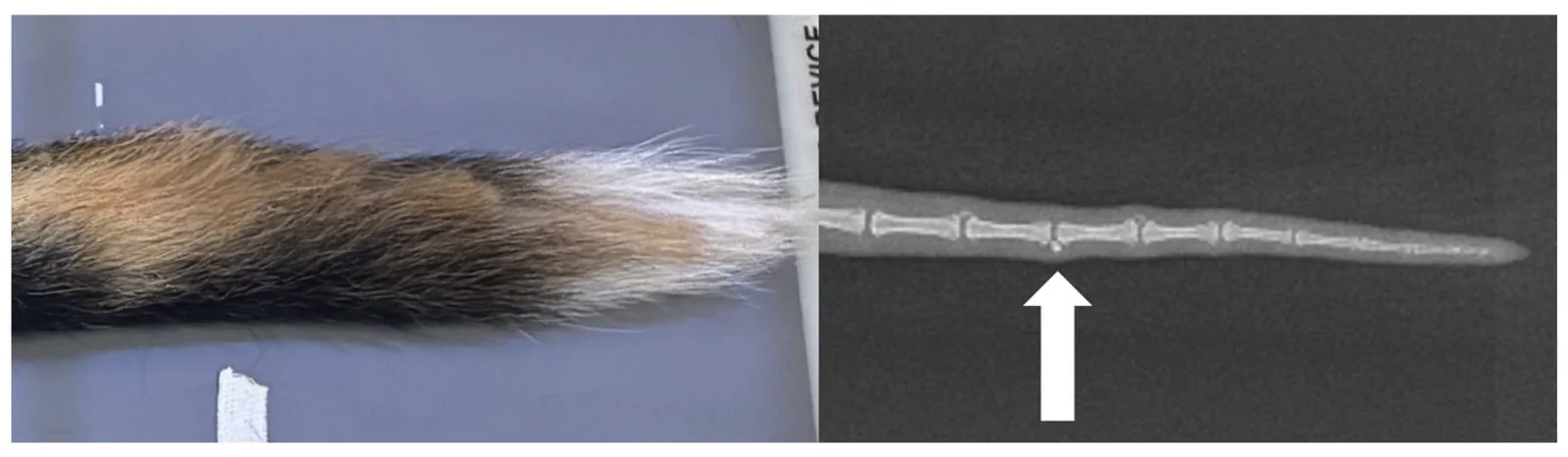
Example of a dog (#45 Vin) exhibiting mineralization (degenerative changes) of the caudal vertebrae within intervertebral spaces: the external view (**left**) and the corresponding radiograph (**right**) are shown.

Of the radiographed population, 47.2% (25/53) of dogs were documented to have a congenital anomaly (block vertebrae, brachyury, and/or hemivertebrae) within their caudal vertebral column (Table 2). One dog exhibited brachyury, with only 14 total caudal vertebrae, and one dog exhibited hemivertebrae (Figure 3). Mild congenital vertebral anomalies (both block vertebrae and hemivertebrae) were detected on radiographs in 7.6% (4/53) of dogs with no abnormalities on physical examination. Additionally, 5.7% (3/53) of the radiographed population were noted to have small bone fragments between intervertebral spaces (Figure 4), indicative of degenerative changes or vertebral mineralization [1]. Because these bone fragments are not considered congenital, these dogs were phenotyped as normal. The Cobb angles ranged from 23.7° to 127.4°, with a median of 48.1°. All dogs with block vertebrae (23/23; 100%) also exhibited axial deviation of the tail.

### 3.2. Studied Parameters and Frequencies

The frequency of block vertebrae within this population was 43.4% (23/53), with 23 dogs having at least two adjacent vertebrae that lacked visible intervertebral spaces identified in their radiographs (Table 2). The median number of block vertebrae within the group of affected dogs was 4 (IQR 2–5). The median angle of the first and second bend in the tail was 47.7 (IQR 36.8–60.4) and 58.0 (IQR 51.4–67.7) degrees, respectively.

Out of the 25 dogs affected, 72.0% (18/25) had deformities in the caudal third of the tail, while 24% (6/25) had deformities in both the middle and caudal third. One dog had a deformity exclusively in the middle third of the tail. Of the 53 dogs radiographed, 24 of them were female and 29 were male (Table 3).

**Figure 5:**
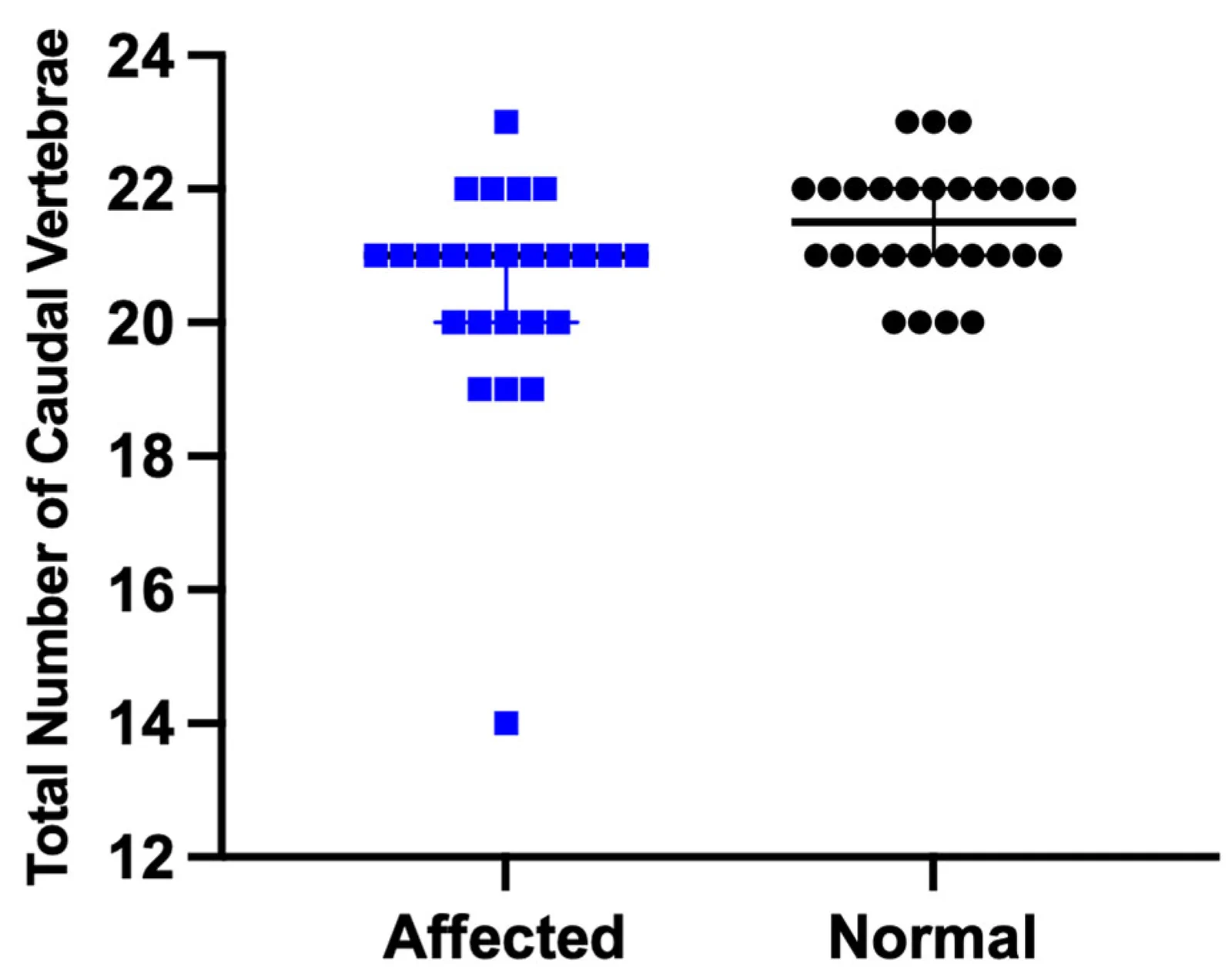
Boxplot graph of the total number of caudal vertebrae in affected (*n* = 25) vs. normal dogs (*n* = 28). The outlier is at 14 vertebrae in the affected group (Figure 3c; #21 UFO).

The median number of caudal vertebrae in this population was 21 (IQR 21–22), with affected dogs having a median of 21.0 (IQR 20–21) and normal dogs having a median of 21.5 (IQR 21–22) (Figure 5).

### 3.3. Scoring Assessment

Of the 25 dogs affected, 36.0% (9/25) were mild, 48.0% (12/25) were moderate, and 16.0% (4/25) were severe (Table 4).

### 3.4. Non-Radiographed Dogs

The non-radiographed population had a median age of 3.9 years (IQR = 1.4–3.3) compared to the radiographed group with a median of 2.5 years (IQR = 3.4–5.9), reflecting their age at adoption or death. However, this does not reflect the time of onset of the abnormalities since visual and palpable tail abnormalities were initially noted at birth. In this group, based on palpation alone, 6/14 (42.8%) dogs had caudal vertebral abnormalities. Since radiographs were not obtained, this percentage could be higher.

### 3.5. Pedigree

**Figure 6:**
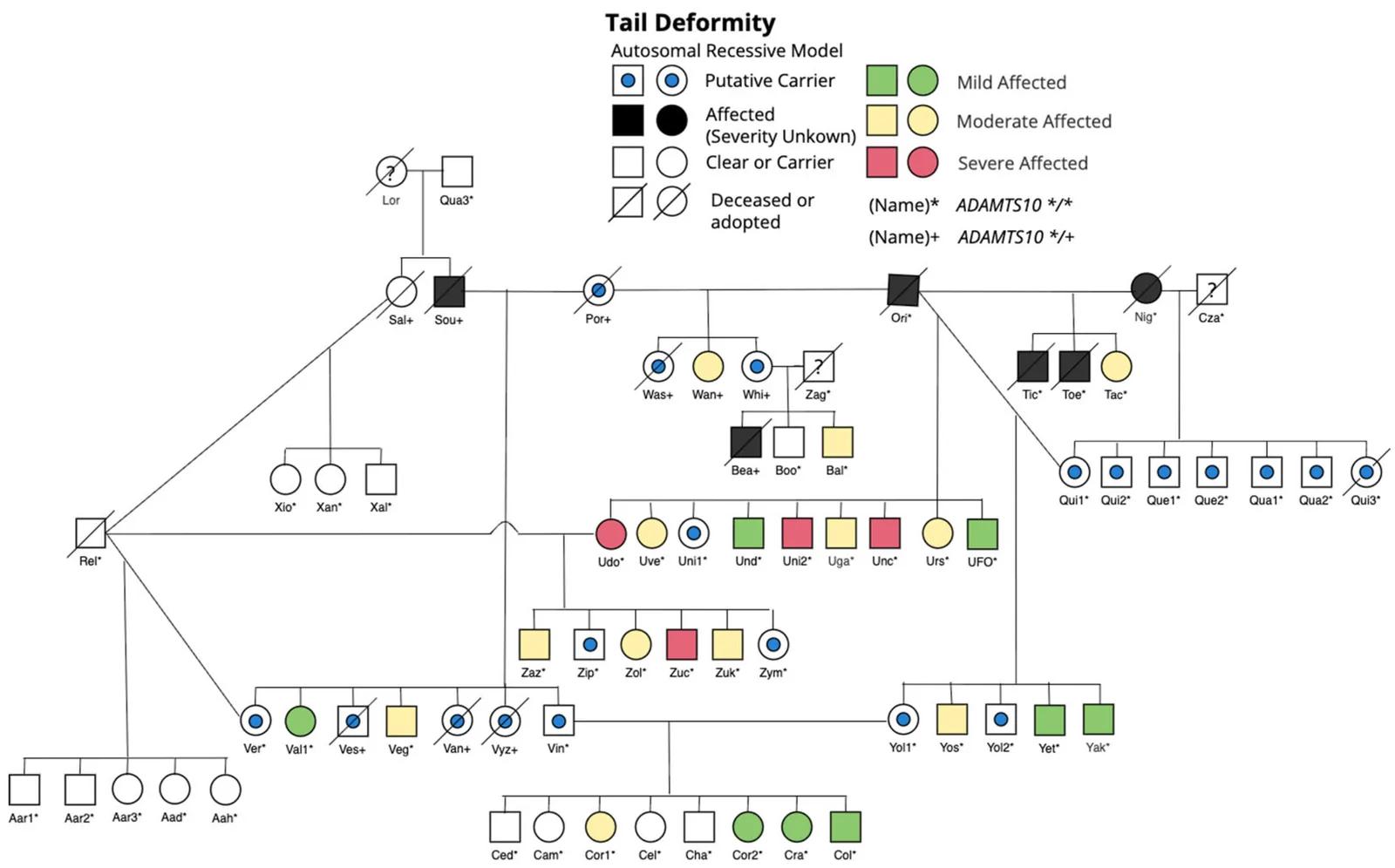
Pedigree of Beagles segregating the tail deformity phenotype (*n* = 67). The pedigree is consistent with a potential autosomal recessive mode of inheritance. Affected animals demonstrate a spectrum of phenotypic severity, classified as mild (green), moderate (yellow), or severe (red). Some of the dogs assessed by physical examination alone, without radiographic evaluation, and thus their phenotypic severity could not be determined, are indicated (black). Three dogs had an unknown tail phenotype and are denoted with a question mark symbol. Putative obligate carriers, inferred on the basis of the suspected autosomal recessive inheritance model, are indicated by concentric symbols. Genotypes for the *ADAMTS10* variant associated with open-angle glaucoma (OAG), which segregates independently within this Beagle colony, are also indicated: ADAMTS10*^/^*, homozygous (OAG affected), and ADAMTS10*^/+^, heterozygous carrier (OAG unaffected) of the G661R missense mutation in *ADAMTS10*.

Pedigree analysis demonstrated segregation of the tail-deformity phenotype in both male and female littermates and throughout the extended pedigree (Figure 6). For pedigree analysis, the tail-deformity phenotype was defined as the presence of a caudal vertebral abnormality identified by physical examination (*n* = 14) and/or radiographic examination (*n* = 53). Affected dogs were also identified among offspring of phenotypically unaffected parents. Among affected littermates for whom severity could be classified, the phenotype showed variable expressivity, ranging from mild to moderate to severe. Under a simple Mendelian model, the observed segregation pattern was consistent with autosomal recessive inheritance, based on the occurrence of affected offspring from unaffected parents and the presence of the phenotype in both males and females [30]. Putative obligate carriers were therefore identified and indicated in the pedigree. Notably, the tail-deformity phenotype segregated independently of the *ADAMTS10* variant associated with primary open-angle glaucoma in Beagles, for which this colony was originally purpose-bred, as confirmed through patterns observed in the pedigree.

## 4. Discussion

In the present study, 47.2% (25/53) of radiographed dogs exhibited at least one congenital caudal vertebral anomaly. Meanwhile, the median number of caudal vertebrae in this population was consistent with the previous literature [1,15]. Anomalies were divided into three groups (block vertebra, hemivertebra, and brachyury) based on radiographic examination findings. Block vertebrae concurrent with axial deviation were the most common finding, presenting in 43.4% (23/53) of the radiographed population, often resulting in a moderate to severe caudal vertebral abnormality score. This prevalence of block vertebrae is higher than in previous studies done in sheep with undocked tails and multiple breeds of dogs, which have published a frequency of 13.0% (28/216) and 22.7% (15/66), respectively [1,26]. The high frequency observed in the present population is notable because block vertebrae have been shown to result from incomplete segmentation of vertebral bodies during development [8]. Although the present study was not designed to determine inheritance, the frequency of block vertebrae within this purpose-bred family of Beagles further provides justification for future genetic investigations. Affected dogs, including one dog with brachyury, had fewer caudal vertebrae compared with normal dogs; however, further investigation is needed to determine whether the vertebral number is related to specific types of caudal vertebral abnormalities or represents normal anatomical variation within the population.

Brachyury and hemivertebrae were much less common findings in this population, with one dog exhibiting each phenotype. The dog with brachyury had only 14 caudal vertebrae, which is substantially fewer than the mean of the population. Brachyury is particularly relevant to future genetic investigations because mutations in exon 1 of the *TBXT* gene have been associated with naturally occurring short tail phenotypes in multiple dog breeds, including the Boxer, Pembrooke Welsh Corgi, and Swedish Vallhund [10,11]. A frameshift deletion variant in the Wnt pathway gene *DVL2* has been associated with brachycephalic phenotypes and caudal vertebral anomalies in English Bulldogs, French Bulldogs and Boston Terriers, while *Pax1* and *Wnt-3a* have been associated with abnormal vertebral development in mouse models [19,20,21]. The past literature has revealed congenital block vertebrae and intervertebral disc protrusion in a young cat [31]. Furthermore, mutations in *T* alleles cause short-tailed phenotypes in the American Bobtail and Pixie-Bob [32]. However, these genes are unlikely to cause congenital anomalies seen in this population due to the normal facial and skeletal conformation observed. While there are multiple caudal phenotypes within the current family of Beagles, future genetic analysis can inform whether these abnormalities share a genetic basis or represent separate conditions.

Importantly, four dogs (7.6%) in the group of radiographed dogs (n = 53) had mild congenital abnormalities identified on radiographs that were not detected during physical examination. This finding is consistent with the previous literature reporting that caudal vertebral anomalies can occur without an obvious kink or axial deviation [2]. Therefore, visual and physical examination alone may underestimate the true prevalence of congenital caudal vertebral anomalies. This is particularly relevant for breeding programs in which the external appearance of the tail may be used to make breeding decisions because a dog with a phenotypically normal tail on physical examination could have an underlying caudal vertebral abnormality. Similarly, accurate identification of affected and unaffected animals is important for future genetic studies because misclassification of affected or unaffected control dogs could complicate or misguide the identification of causal genetic variants.

The location of the abnormalities in the affected dogs in the present study was exclusive to the middle and caudal third of the tail, which agrees with the previous literature published in dogs and sheep with undocked tails [1,26]. Of the affected dogs, a majority had deformities involving the caudal third of the tail. This concentration towards the distal area of the tail may contribute to the difficult detection of deformities externally. Caudal vertebrae get progressively smaller and shorter towards the tip of the tail. Therefore, smaller distal vertebrae may exhibit abnormalities that can only be identified radiographically, as they may not demonstrate a visible or palpable axial deviation. However, this mechanism was not directly evaluated in the present study and represents a possible explanation rather than a demonstrated mechanism.

Previous classifications of caudal vertebral abnormalities have focused primarily on identifying and categorizing the type of abnormality present. For example, Paninárová et al. [1] classified caudal vertebral abnormalities based on radiographic appearance and etiology. The main reason for this classification was to distinguish congenital, morphological, post-traumatic, and degenerative changes from each other. However, this classification did not provide a method for grading the overall severity of caudal vertebral abnormalities within an individual dog. The scoring system developed in the present study builds upon the phenotypic characterization of these abnormalities by incorporating the extent of vertebral fusion, degree of axial deviation, and presence of brachyury and hemivertebrae into a standardized severity classification. Although the proposed mild, moderate, and severe categories have not been clinically validated, they provide a consistent method for describing the spectrum of caudal vertebral phenotypes observed within this population.

Autosomal recessive traits affect males and females similarly and may appear among siblings born to unaffected carrier parents. Importantly, the tail deformity phenotype was not uniform among affected dogs. Where radiographic characterization was available, caudal vertebral anomalies ranged from mild to moderate to severe, including among littermates. This variability may reflect variable expressivity of a phenotype among individuals with the same underlying genetic condition, potentially influenced by additional genetic modifiers, environmental factors, or both. However, because a shared underlying genetic cause has not been established, variable expressivity remains a proposed explanation for the observed variation in severity. Taken together, the observed pedigree structure and equal distribution between sexes is consistent with autosomal recessive inheritance as the most parsimonious working hypothesis. Nevertheless, this conclusion remains provisional pending molecular characterization, particularly because the phenotype may represent a continuous trait rather than a strictly binary affected/unaffected condition. Additionally, because not all pedigree members were radiographically phenotyped, the proposed mode of inheritance should be considered preliminary and does not establish a defined Mendelian inheritance pattern. Notably, all dogs with abnormal tails carry the *ADAMTS10* G661R missense mutation, which can affect the musculoskeletal system [33]. Weill–Marchesani syndrome (WMS) is a rare condition that is characterized by the association of short stature, brachydactyly (abnormally short fingers and toes), joint stiffness, and eye anomalies [33]. WMS can be conclusively diagnosed by the identification of homozygous pathogenic variants in *ADAMTS10* [31]; however, it does not seem to play a role in the tails of the dogs in the present study since they do not exhibit any of the characteristics commonly seen in WMS patients. The presence of phenotypically unaffected dogs carrying the variant indicates that the *ADAMTS10* genotype does not co-segregate with the tail-deformity phenotype within this pedigree. Therefore, we found no indication that the abnormal tails are associated with the *ADAMTS10* mutation. Genetic discovery studies should therefore incorporate detailed phenotyping, including radiographic severity scoring, and prioritize comparison of clinically normal dogs with dogs at the severe end of the phenotypic spectrum. Greater phenotypic contrast may improve the ability to identify genetic variants associated with the tail-deformity phenotype while reducing uncertainty associated with mildly affected individuals.

The detection of a kink tail on palpation is a reason that breeders may exclude a dog from breeding based on individual club regulations. However, this study and the previous literature have revealed that some caudal vertebral deformations are not able to be detected through physical examinations alone [1,2]. The previous literature has also reported that many vertebral anomalies do not produce clinical symptoms and are detected only as incidental findings on radiographs of neurologically intact animals [10]. Another study documented a high number of block vertebrae and hemivertebrae in brachycephalic breeds, which have been selectively bred to have a screw tail trait [17]. Hemivertebrae and block vertebrae in the caudal vertebral column are suspected to be heritable [2]. Therefore, accurately identifying these abnormalities is especially important when considering their potential heritability.

There were several limitations to this study. Most notably, while 53 of the 67 dogs in the pedigree could be radiographically examined, the remaining 14 dogs were either adopted or deceased and were classified based on physical examination and records alone, meaning we may have underestimated the number of affected dogs in this group. This incomplete radiographic phenotyping may have resulted in misclassification of unaffected dogs, potentially influencing the pedigree-based segregation analysis and proposed mode of inheritance. The Cobb angle method used to measure tail angulation in the present study has not been validated for caudal vertebrae, as it has primarily been used to quantify spinal curvature and deformities in the thoracic region of humans and dogs [25,26]. Therefore, its application to caudal vertebral anomalies may introduce measurement variability, and further validation is needed to establish its reliability and reproducibility in assessing tail angulation. Furthermore, the present study was limited to descriptive statistics given the nature of the study and the inclusion of dogs from a single pedigree. Additionally, no molecular genetic analysis was performed, and the proposed mode of inheritance therefore remains unconfirmed. Furthermore, the prevalence of block vertebrae reported in this study is higher than previously reported in studies of sheep with undocked tails and multiple breeds of dogs [1,24]. However, direct comparison of prevalence among these studies should be interpreted cautiously because of differences in species, breed composition, study populations, and sampling methods used to identify and classify caudal vertebral abnormalities. Finally, because this study examined a single purpose-bred population, these findings should not be generalized to the overall Beagle breed without additional study.

## 5. Conclusions

In conclusion, establishing an accurate phenotype through the present study provides an important foundation for further investigation to determine the genetic basis of caudal vertebral anomalies in Beagles. Genetic analysis could compare affected and unaffected dogs and investigate genes known to influence vertebral and axial development. The identification of radiographic abnormalities in dogs with no detectable abnormalities on physical examination emphasizes the importance of radiographic evaluation for accurate phenotypic classification. Ultimately, this study provides a detailed phenotypic characterization of caudal vertebral anomalies within the radiographically examined Beagle population and demonstrates the importance of radiographic evaluation for accurate identification and classification of these abnormalities.

## Figures and Tables

**Table 1:** Novel scoring system for severity of canine caudal vertebral abnormalities (mild = 1–2 points; moderate = 3–4 points; severe = 5–7 points).

**Number of Fused (Block) Vertebrae**	**Point(s) Gained**
0–1	0
2–3	1
4–8	2
9+	3
**Angle of Bend(s)**	**Point(s) Gained**
0°	0
>0° to 60°	1
>60°	2
**Type of Deformity**	**Point(s) Gained**
brachyury	1
hemivertebrae	1

**Table 2:** Prevalence of specific types of anomalies within the population (*n* = 53).

Congenital Anomaly Type	*n*	Pop%	Anm%
Block vertebrae	23	43.4 (23/53)	92.0 (23/25)
Brachyury	1	1.9 (1/53)	4.0 (1/25)
Hemivertebrae	1	1.9 (1/53)	4.0 (1/25)

*n*: number; Pop%: percentage of population (*n* = 53); Anm%: percentage of dogs affected (*n* = 25).

**Table 3:** Absolute frequencies of affected and normal female (*n* = 24) and male (*n* = 29) dogs that were phenotypically confirmed through radiographic examination (*n* = 53).

Gender	Affected	Normal
Female	10/24 (41.7%)	14/24 (58.3%)
Male	15/29 (51.7%)	14/29 (48.3%)

**Table 4:** List of information and findings of dogs that were evaluated by radiography (*n* = 53). An x is denoted for an absence of a category.

Study ID#	Dog Name	Caudal Vertebrae Status	Total Caudal Vertebrae	Total Block Vertebrae	Location of Deformity	Congenital Anomaly Type	Cobb Angle (°)
1	Udo	Affected (severe)	20	5	Cd13–Cd17	Block vertebrae	67.0; 39.0
2	Unc	Affected (severe)	21	4	Cd14–Cd17	Block vertebrae	37.5; 72.1
3	Uni2	Affected (severe)	20	8	Cd12–Cd19	Block vertebrae	77.6; 59.0
4	Zuc	Affected (severe)	20	8	Cd13–Cd20	Block vertebrae	71.7; 87.2
5	Veg	Affected (moderate)	21	5	Cd14–Cd18	Block vertebrae	44.3; 57.0
6	Uve	Affected (moderate)	21	4	Cd13–Cd16	Block vertebrae	23.7; 41.4
7	Zaz	Affected (moderate)	21	6	Cd13–Cd18	Block vertebrae	106.6
8	Bal	Affected (moderate)	19	6	Cd14–Cd19	Block vertebrae	47.6
9	Cor1	Affected (moderate)	21	5	Cd15–Cd19	Block vertebrae	53.0
10	Tac	Affected (moderate)	23	5	Cd16–Cd20	Block vertebrae	59.6
11	Uga	Affected (moderate)	21	2	Cd17–Cd18	Block vertebrae	62.8
12	Urs	Affected (moderate)	22	3	Cd14–Cd16	Block vertebrae	48.0; 54.7
13	Wan	Affected (moderate)	19	3	Cd14–Cd16	Block vertebrae	127.4
14	Yos	Affected (moderate)	21	5	Cd14–Cd18	Block vertebrae	43.4; 66.2
15	Zol	Affected (moderate)	21	6	Cd13–Cd18	Block vertebrae	24.6
16	Zuk	Affected (moderate)	21	5	Cd13–Cd17	Block vertebrae	51.6
17	Col	Affected (mild)	22	2	Cd16–Cd17	Block vertebrae	52.9
18	Cor2	Affected (mild)	22	0	Cd20–Cd22	Hemivertebrae	37.8
19	Cra	Affected (mild)	20	2	Cd19–Cd20	Block vertebrae	29.1
20	Ott	Affected (mild)	19	2	Cd15–Cd16	Block vertebrae	33.6
21	UFO	Affected (mild)	14	0	x	Brachyury	x
22	Und	Affected (mild)	21	2	Cd15–Cd16	Block vertebrae	47.7
23	Val	Affected (mild)	22	3	Cd15–Cd17	Block vertebrae	40.1
24	Yak	Affected (mild)	20	2	Cd7–Cd8	Block vertebrae	28.6
25	Yet	Affected (mild)	21	2	Cd16–Cd17	Block vertebrae	34.6
26	Aad	Normal	23	0	x	x	x
27	Aah	Normal	23	0	x	x	x
28	Aar1	Normal	23	0	x	x	x
29	Aar3	Normal	22	0	x	x	x
30	Boo	Normal	22	0	x	x	x
31	Cam	Normal	22	0	x	x	x
32	Ced	Normal	22	0	x	x	x
33	Cel	Normal	22	0	x	x	x
34	Cha	Normal	21	0	x	x	x
35	Osc	Normal	21	0	x	x	x
36	Qua1	Normal	21	0	x	x	x
37	Qua2	Normal	21	0	x	x	x
38	Qua3	Normal	22	0	x	x	x
39	Que1	Normal	22	0	x	x	x
40	Que2	Normal	20	0	x	x	x
41	Qui1	Normal	21	0	x	x	x
42	Qui2	Normal	21	0	x	x	x
43	Uni1	Normal	20	0	x	x	x
44	Ver	Normal	20	0	x	x	x
45	Vin	Normal	21	0	x	x	x
46	Whi	Normal	20	0	x	x	x
47	Xal	Normal	21	0	x	x	x
48	Xan	Normal	22	0	x	x	x
49	Xia	Normal	22	0	x	x	x
50	Yol1	Normal	22	0	x	x	x
51	Yol2	Normal	21	0	x	x	x
52	Zip	Normal	22	0	x	x	x
53	Zym	Normal	21	0	x	x	x

## Data Availability

The data that support the findings of this study are available from the corresponding author upon reasonable request.

## Abbreviations

The following abbreviations are used in this manuscript:

DICOM	Digital Imaging and Communications in Medicine
*DVL1*	*Dishevelled segment polarity protein 1* gene
*DVL2*	*Dishevelled segment polarity protein 2* gene
*DVL3*	*Dishevelled segment polarity protein 3* gene
*ADAMTS10*	*ADAM metallopeptidase with thrombospondin type 1 motif 10* gene
*TBXT*	*T-box transcription factor T* gene
*Pax1*	*Paired Box Gene 1* gene
Wnt	Wingless-related integration site
*Wnt-3a*	*Wingless*-*type MMTV integration site family member 3A* gene
*HES7*	*Hes family bHLH transcription factor 7* gene
WMS	Weill–Marchesani syndrome
